# Protective Effect of Glycomacropeptide on the Inflammatory Response of U937 Macrophages

**DOI:** 10.3390/foods12071528

**Published:** 2023-04-04

**Authors:** Laura Elena Córdova-Dávalos, Daniel Cervantes-García, Maria Fernanda Ballona-Alba, Alejandra Santos-López, Alma Saraí Esquivel-Basaldúa, Pamela Gallegos-Alcalá, Mariela Jiménez, Eva Salinas

**Affiliations:** 1 Laboratory of Immunology, Department of Microbiology, Center of Basic Science, Universidad Autónoma de Aguascalientes, Av. Universidad # 940, Aguascalientes 20100, Mexico; laura.cordova@edu.uaa.mx (L.E.C.-D.); or dcervantesga@conacyt.mx (D.C.-G.); ferchis_ballo@hotmail.com (M.F.B.-A.); ale.liz.salo@gmail.com (A.S.-L.); saraeba102@gmail.com (A.S.E.-B.); pamela.g.alcala@gmail.com (P.G.-A.); mayojv@hotmail.com (M.J.); 2 National Council of Science and Technology, Av. de los Insurgentes Sur 1582, Crédito Constructor, Benito Juárez, Ciudad de México 03940, Mexico

**Keywords:** glycomacropeptide, sialic acid, human macrophage, inflammation, oxidative stress, SOCS3, HMOX-1

## Abstract

Macrophages play crucial roles in inflammation and oxidative stress associated with noncommunicable diseases, such as cardiovascular diseases, diabetes, and cancer. Glycomacropeptide (GMP) is a bioactive peptide derived from milk κ-casein that contains abundant sialic acid and has shown anti-inflammatory, antioxidative, anti-obesity, and anti-diabetic properties when is orally administered. The aim of this study was to evaluate the effect of GMP on the regulation of the inflammatory response in human macrophages and the participation of sialic acid in this activity. GMP pretreatment decreased by 35%, 35%, and 49% the production of nitrites, interleukin (IL)-1β, and tumor necrosis factor (TNF)-α, respectively, in activated human macrophages U937. The same effect was obtained when cells were pretreated with asialo GMP, and no change on the gene expression of the lectins associated with the recognition of sialic acids, *SIGLEC5*, *7,* and *9,* was induced by GMP on macrophages, which suggests that sialic acid might not be involved in this immunoregulatory effect. Interestingly, GMP increased 8.9- and 3.5-fold the gene expression of the canonical anti-inflammatory protein *SOCS3* and the antioxidant enzyme *HMOX1*, respectively, in U937 cells. Thus, GMP exerts anti-inflammatory and antioxidative activities on activated macrophages in a sialic acid-independent manner, which might be related to its in vivo reported bioactivity.

## 1. Introduction

Nowadays, more than 70% of global deaths are due to noncommunicable diseases, mainly cardiovascular diseases, diabetes, cancer, and chronic respiratory diseases [1]. Noncommunicable diseases are highly favored by smoking, sedentary lifestyles, and the Western diet, characterized by the high consumption of saturated fatty acids, salt, and refined sugar, which contributes to a dysfunction of the immune system and obesity [2]. The latter causes increased lipid storage in adipose tissue that affects its endocrine and regulatory functions and triggers a state of low-grade inflammation [3]. Macrophages are the main immune cells involved in this inflammatory process [4]. In obesity, adipose tissue-resident macrophages change their phenotype from M2 toward M1, which increases the production of pro-inflammatory cytokines, such as tumor necrosis factor (TNF)-α [5]. Consequently, there is lipolysis and the release of saturated fatty acids in hypertrophied adipocytes, which activate macrophages through nuclear factor-κB (NF-κB)-dependent mechanisms to induce the expression of more proinflammatory genes, such as inducible nitric oxide synthase (iNOS) and interleukin (IL)-6 [4,6]. Moreover, several studies have shown a tight association between TNF-α and insulin resistance in obesity [7,8,9]. The inflammatory response of macrophage can be regulated in different ways, among which the regulation of suppressor of cytokine signaling (SOCS)3 expression appears to have a crucial role [10] as SOCS proteins are critical to the normal functioning and cessation of the primary cytokine signal [11].

It is currently known that food is a source of diverse active compounds that can improve human health, such as bioactive peptides [12]. The glycomacropeptide (GMP) is a milk bioactive peptide which is generated from hydrolyzing κ-casein by chymosin during cheese making or by pepsin during gastric digestion [13,14,15,16]. It is a peptide of 64 amino acids which is O-glycosylated in threonine and serine. GMP oligosaccharides are formed by galactosyl, N-acetylgalactosamine, and sialic acid, the latter being the prominent terminal sugar unit of GMP, linked to N-acetylgalactosamine by α-2,6 bond and to galactosyl by α-2,3 bond [17,18,19]. Some of the biological activities of GMP are mediated by the peptide chain, while other bioactivities have been associated to sialic acid [20]. GMP was found to exert immunoregulatory, anti-inflammatory, and antioxidant activities in models of allergy and intestinal inflammatory diseases [21,22,23,24,25,26,27]. In relation to metabolic diseases, GMP intake has shown promising effects on the prevention of insulin resistance and liver dysmetabolism in mice receiving a high-fat and high-sucrose diet [28]. In a streptozotocin-induced and high-fat diet-induced diabetic mouse model, a papain hydrolyzed GMP showed antidiabetic effects [29]. Moreover, in vitro and in vivo assays have demonstrated that GMP reduces lipid accumulation in adipocytes, body weight gain, and hyperlipidemia in obese rats [30,31]. The effect of GMP in obesity and metabolic diseases has been partly associated with the regulation of gut microbiota and oxidant and inflammatory processes [28,29,31,32]. Whether GMP is modulating macrophage inflammatory response still needs to be clarified.

There are few reports about the effect of GMP on macrophages activation. The only study developed in human macrophages showed that GMP exerts immunomodulatory effects on macrophage-differentiated U937 cells by increasing their proliferation and phagocytic activity. Interestingly, the sialic acid moieties play important roles in this immunoenhancing effect [20]. In lipopolysaccharide (LPS)-activated RAW264.7 murine macrophages, GMP and its papain hydrolysate downregulate nitrite formation and iNOS, TNF-α, and IL-1β mRNA expression through the modulation of NF-κB signaling pathway [33]. In the same in vitro model, an antioxidant effect is also attributed to GMP that is mediated by heme oxygenase-1 (HMOX-1) expression [34], a molecule extensively involved in anti-inflammatory and antioxidative processes [35].

Collectively, these data suggest that the modulation of the inflammatory response of human macrophages by GMP might be beneficial in those pathological conditions in which their continuous activation contributes to the pathogenesis or to the development of complications. Thus, in the current study, we investigated the preventive effect of GMP on inflammatory response of the U937-differentiated human macrophages. The participation of sialic acid in this effect was evaluated using asialo GMP (aGMP). Additionally, some molecules involved in GMP immunoregulatory activity were identified.

## 2. Materials and Methods

### 2.1. Production of Asialo GMP

GMP used in this study (Lacprodan cGMP-10) was gifted by Arla Food Ingredients Group P/S (Viby, Denmark). To obtain aGMP, increasing concentrations of neuraminidase (sialidase) from Vibrio cholerae (0.05, 0.1, 0.2 U; Roche-Sigma Aldrich-Merck, Mannheim, Germany) was added to GMP (2.5 µg/µL) in 20 mM sodium acetate, pH 5.5, at a final volume of 20 µL. Samples were incubated at 37 °C overnight. The enzyme was inactivated heating at 85 °C for 15 min. Free sialic acids were removed by dialysis with a 0.5–1 kDa cut-off membrane (Biotech, Somerset, NJ, USA).

### 2.2. Electrophoresis and Western Blot

The electrophoretic separation of protein sample was developed by SDS-PAGE [36] using the Bio-Rad Miniprotean III System (Bio-Rad, Hercules, CA, USA). The elimination of sialic acid from GMP was verified by western blot using either biotinylated-lectins from *Sambucus nigra* (SNA) or *Maackia amurensis* II (MAL II), which bind preferentially to sialic acid α-2,6 or α-2,3 linked, respectively (both lectins from Vector Laboratories, Newark, CA, USA). Briefly, proteins were electro-transferred from the gel onto polyvinylidene difluoride membranes at 12 mAmp, overnight. Membranes were blocked with 5% bovine serum albumin (BSA) in Tris buffer (TBS-BSA) for 3 h at room temperature (RT) and incubated with biotinylated-SNA 4 µg/mL or biotinylated-MAL II 1 µg/mL for 1 h at RT. After three washes with 0.1% Tween-20 in TBS (TTBS), membranes were incubated with streptavidin-HRP (1:25,000) diluted in TBS-BSA for 2 h at RT. As the control, GMP was detected in a duplicated membrane incubated with a rabbit polyclonal anti-GMP (1:500) overnight at 4 °C and later with HRP-conjugated anti-rabbit secondary antibody (1:5000) 2 h at RT [37]. After washing three times with TTBS, HRP activity was detected in membranes with the Clarity^TM^ Western ECL substrate (Bio-Rad, Hercules, CA, USA), and images were acquired with the Microchemi 4.2 chemiluminescence system (DNR Bio-Imaging Systems, Modi’in-Maccabim-Re’ut, Israel).

### 2.3. Monocyte Culture, Differentiation, and Stimulation

The U937 monocytes (ATCC^®^ CRL-1593.2TM; Manasas, VA, USA) were cultured in RPMI-1640 with L-glutamine, supplemented with 10% inactivated fetal bovine serum (FBS; Gibco, Carlsbad, CA, USA), 100 U/mL penicillin, and 100 μg/mL streptomycin in a 5%-CO_2_ humidified incubator at 37 °C. At a density of 1 × 10^6^ cells/mL per well, U937 monocytes were differentiated to macrophage with 100 nM Phorbol 12-myristate 13-acetate (PMA; Sigma-Aldrich, St. Louis, MO, USA) for 72 h in 24-well culture plates. The medium with PMA was replaced by fresh medium, and the cells were incubated for 24 h. Macrophages were pretreated with GMP or aGMP prepared in supplemented medium at indicated concentrations for 12 h at 37 °C. Then, cells were stimulated with LPS (Sigma-Aldrich) from *Escherichia coli* O111:B4 (10 µg/mL) in the presence of 20 ng/mL of interferon-γ (IFN-γ; PeproTech, Cranbury, NJ, USA). Cells without GMP and without LPS+IFN-γ were considered as the control condition (basal secretion). After 24 h incubation, cell-free supernatants were collected for nitric oxide (NO) and cytokine quantification. Cell viability was determined by the 3-(4,5-dimethylthiazol-2-yl)-2,5-diphenyl tetrazolium bromide (MTT, Sigma-Aldrich, St. Louis, MO, USA) technique [38]. Ten microliters of MTT solution (5 mg/mL) were added to each well, and the cells were incubated for 4 h. Then, the purple formazan crystals were solubilized with 0.04 N hydrochloride in isopropyl alcohol and plates were mixed thoroughly and read at 595 nm on a microplate reader (Bio-Rad, Tokyo, Japan). The absorbance of the LPS/IFN-γ group was considered as 100% of cell viability.

### 2.4. Measurement of NO Production

The nitrite accumulated in culture medium was measured as an indicator of NO production in macrophages, based on the Griess reaction system [39]. Briefly, 100 μL of each supernatant was mixed with an equal volume of Griess reagent (1% sulfanilic acid in 5% phosphoric acid; 0.5% α-Naphthylamine in 5N acetic acid). After being incubated at RT for 5 min, the sample absorbance was measured at 540 nm in an iMark plate spectrophotometer (Bio-Rad, Tokyo, Japan). The concentrations of nitrite present in the samples were calculated from a standard curve (0 to 100 µM NaNO_2_) in culture medium.

### 2.5. Quantification of IL-1β and TNF-α

Supernatant samples were collected from each assay and stored at −70 °C until used for cytokine determination. Total IL-1β and TNF-α levels in supernatants were quantified using a human IL-1β ELISA kit (Invitrogen IL-1 beta Human ELISA Kit; Thermo Fisher Scientific, Nivelles, Belgium) or human TNF-α ELISA kit (Invitrogen TNF alpha Human ELISA Kit; Thermo Fisher Scientific, Rockville, MD, USA) according to the manufacturer’s instructions.

### 2.6. RNA Isolation, Reverse Transcription, and Real-Time Quantitative Polymerase Chain Reaction

U937-derived macrophages were seeded at 3 × 10^6^ cells per well in six-well plates, pretreated with GMP, as previously mentioned, and stimulated with 10 µg/mL LPS and 20 ng/mL IFN-γ for 4 h. Total RNA was isolated from cells using TRIzol reagent (TRIzol, Sigma-Aldrich, St. Louis, MO, USA) according to the manufacturer’s instructions. RNA was retrotranscribed to cDNA using the First Strand cDNA Synthesis Kit from Thermo Scientific (San Jose, CA, USA). Real time quantitative PCR (RT-qPCR) was carried out with Applied Biosystem StepOne Real Time PCR system (Thermo Scientific, San Jose, CA, USA) using Maxima SYBR Green/ROX qPCR Master Mix (2X) (Thermo Scientific, San Jose, CA, USA) in 20 μL of reaction volume for sialic acid-binding immunoglobulin-like lectin (*SIGLEC*) 5, 7, and 9, *SOCS3,* and *HMOX1*. Primers used for the quantification of mRNA expression are listed in Table 1. RT-qPCR was performed starting with a denaturation at 95 °C for 3 min followed by 40 cycles of 95 °C for 30 s and 60 °C for 30 s. The 2^−ΔΔCt^ method was used to obtain fold changes in mRNA abundance [40]. Data were normalized by the level of *GAPDH* mRNA expression in each sample and presented as the fold change relative to the control.

### 2.7. Statistical Analysis

All data were expressed as the mean ± standard error of the mean (SEM). Comparisons among groups were made with the one-way analysis of variance (ANOVA) test and Tukey-Kramer multiple comparison post hoc using the GraphPad Prism 8.0 software (GraphPad Software Inc., La Jolla, CA, USA). Significance was set at *p* < 0.05. Groups were marked with different letters (a, b, c, d) if there were significant differences among means and were marked with the same letter if there were not significant differences among means.

## 3. Results

### 3.1. GMP Effect on U937 Macrophage Activation by LPS and IFN-γ

**Figure 1:**
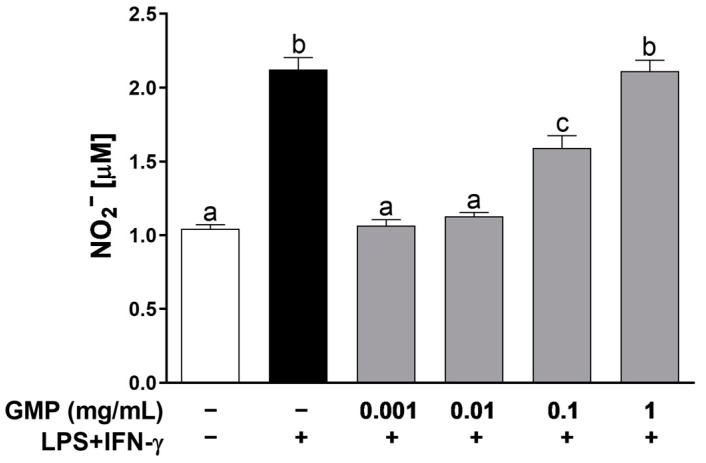
Effect of GMP on NO production by stimulated U937 macrophages. Cells were incubated in the absence or presence of increasing concentrations of GMP for 12 h and later stimulated with LPS (10 µg/mL) and IFN-γ (20 ng/mL) for 24 h (*n* = 9, 3 independent experiments in triplicate). Means followed by different letters (a, b, c) are significantly different.

To determine the activity of GMP on inflammatory response of U937 macrophages, we evaluated the NO-production stimulated by LPS and IFN-γ in cells pretreated with GMP (Figure 1). When cells were stimulated with LPS and IFN-γ, the nitrite concentration in the medium was two-fold higher than in the control conditions. Pretreatment with GMP at concentrations that ranged from 0.001 to 0.1 mg/mL markedly decreased nitrite-stimulated production in comparison with the LPS+IFN-γ group (*p* < 0.001). However, the inhibitory activity was abolished at the dose of 1 mg/mL.

**Figure 2:**
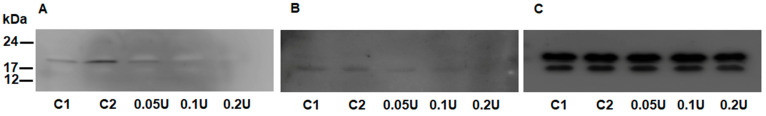
Asialylation of GMP with neuraminidase. The products of GMP-neuraminidase reaction were separated by SDS-PAGE and detected by western blot using: (**A**) SNA lectin, (**B**) MALII lectin, (**C**) or a polyclonal anti-GMP antibody. The amount of neuraminidase used in each reaction is indicated below the lines. C1, intact GMP dissolved in water; C2, intact GMP dissolved in the reaction buffer.

To evaluate sialic acid involvement in the GMP inhibitory effect, sialic acid was removed with neuraminidase to generate aGMP, and the presence of sialic acid before and after GMP neuraminidase treatment was analyzed using lectins. Staining with SNA lectin showed that sialic acid was mostly α-2,6 bound to N-acetil galactosamine in GMP with high molecular weight (band at 20 kDa; Figure 2A), while staining with MAL II detected the presence of sialic acid α-2,3 in both GMP bands, although it was more abundant in that of low molecular weight (band at 14 kDa; Figure 2B). When GMP was incubated with increasing concentrations of neuraminidase, the lectin labelling decreased in a sialidase concentration-dependent manner, indicating that the terminal sialic acids of GMP were removed. Sialic acids were totally removed with 0.2 U/mL of neuraminidase, which allowed for obtaining aGMP. This neuraminidase concentration was used in biological assays. The peptide was equally present in all samples, as detected by immuno-blot using a polyclonal anti-GMP antibody (Figure 2C), showing that the decrease in the staining of the GMP bands was due to the decrease or lack of sialic acid in the GMP molecule and not to a reduced amount of the peptide in the sample.

**Figure 3:**
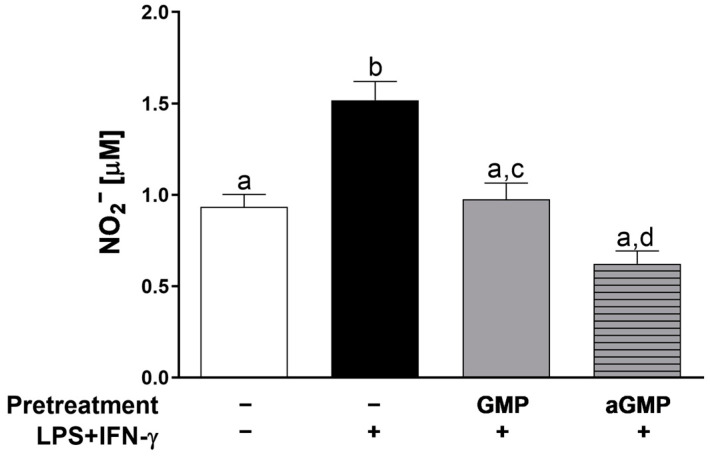
Effect of GMP and aGMP on NO production by stimulated U937 macrophages. Cells were incubated (pretreated) in the absence or presence of GMP (2.5 µg/µL) or aGMP (2.5 µg/µL) for 12 h and later stimulated with LPS (10 µg/mL) and IFN-γ (20 ng/mL) for 24 h (*n* = 9, 3 independent experiments in triplicate). Means followed by different letters (a, b, c, d) are significantly different.

As GMP at 0.001 and 0.01 mg/mL reduced NO-stimulated production to similar levels to the control group, we used the higher GMP concentration to produce aGMP to investigate whether sialic acid was involved in the inhibitory effect. Thus, cells were pretreated with intact or aGMP to compare the effect on NO production. As shown in Figure 3, the production of nitrites in cells pretreated with aGMP (0.62 ± 0.06 µM) was reduced at a slightly and significantly lower level than when intact GMP was used (0.97 ± 0.08 µM), as compared to LPS+IFN-γ group (1.51 ± 0.10 µM).

**Figure 4:**
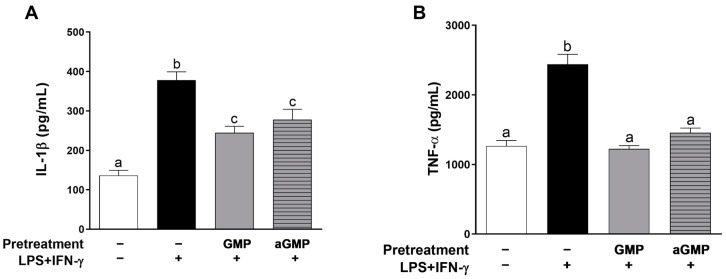
Effect of GMP and aGMP on the secretion of inflammatory cytokine by stimulated U937 macrophages. Cells were incubated (pretreated) in the absence or presence of GMP (2.5 µg/µL) or aGMP (2.5 µg/µL) for 12 h and later stimulated with LPS (10 µg/mL) and IFN-γ (20 ng/mL) for 24 h. (**A**) IL-1β and (**B**) TNF-α were quantified on supernatants by ELISA (*n* = 6, 2 independent experiments in triplicate). Means followed by different letters (a, b, c) are significantly different.

The inflammatory response of macrophages is characterized by the secretion of proinflammatory cytokines [41]. Thus, we also evaluated the effect of GMP and aGMP on IL-1α and TNF-β release (Figure 4). The stimulation of the cells with LPS+IFN-γ increased 2.7-fold (378 ± 21.26 pg/mL) and 1.9-fold (2438 ± 144.9 pg/mL) the secretion of IL-1β and TNF-α as compared to basal secretion (136.3 ± 13.03 pg/mL and 1266 ± 78.04 pg/mL). GMP and aGMP pretreatments inhibited stimulated secretion of both cytokines by 35% and 26%, respectively, to IL-1β (244.7 ± 16.48 pg/mL and 278 ± 26.06 pg/mL) and 49% and 40%, respectively, to TNF-α (1225 ± 44.98 pg/mL and 1456 ± 65.97 pg/mL). However, there was no difference on the secretion of IL-1β and TNF-α between GMP and aGMP, indicating that sialic acid was not involved in the anti-inflammatory effect.

To rule out the possibility that the inhibition of the inflammatory response of U937 macrophages was due to the cytotoxic effect of GMP or aGMP, cell viability was evaluated. As shown in Table 2, the percentages of cell viability in the GMP group and aGMP group were not significantly different from that of the LPS/IFN-γ group, showing that GMP experimental concentrations did not alter cell viability.

### 3.2. Effect of GMP on the Gene Expression of Siglec Receptors

**Figure 5:**
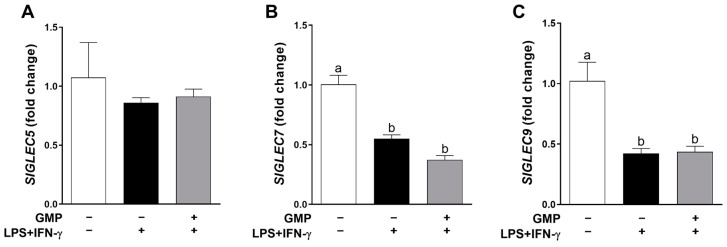
Effect of pretreatment with GMP on macrophage expression level of Siglec receptors. Expression level of (**A**) *SIGLEC5*, (**B**) *SIGLEC7*, and (**C**) *SIGLEC9* receptors relative to *GAPDH* was analyzed by quantitative PCR and expressed as fold change (*n* = 3 independent experiments). Means followed by different letters (a, b) are significantly different.

To evaluate whether the inhibitory activity of GMP on inflammatory activation of U937 macrophages is mediated by the up-regulation of inhibitory receptors, we analyzed mRNA levels of three Siglec receptors, which are related to the dampening of the inflammatory response and have been reported in human macrophages that recognize α-2,3 or α-2,6 sialic acid as ligands [42]. As shown in Figure 5, macrophage activation by LPS+IFN-γ did not change *SIGLEC5* expression but remarkably reduced the gene expression of *SIGLEC7* by 45% (*p* < 0.01) and *SIGLEC9* by 58% (*p* < 0.05). Pretreatment with GMP did not modify *SIGLEC5*, *7*, and *9* expression level under the inflammatory stimulus, suggesting that the inhibitory action on macrophage activation is exerted in an independent-Siglec manner.

### 3.3. Regulatory Effect of GMP on SOCS3 and HMOX1 Expression

**Figure 6:**
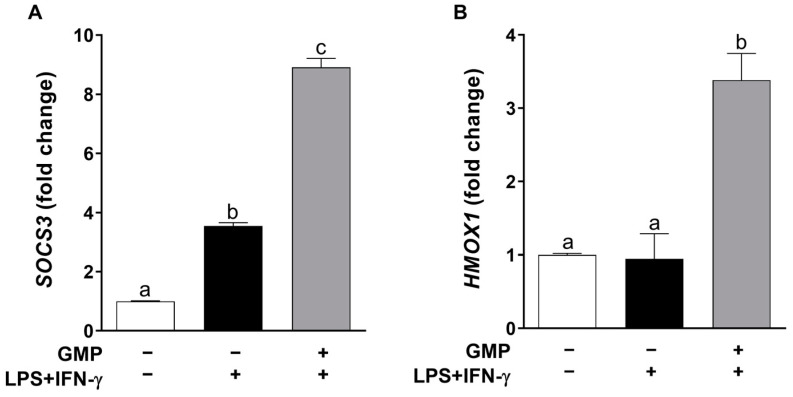
Effect of pretreatment with GMP on macrophage expression level of regulatory molecules. Expression levels of (**A**) *SOCS3* and (**B**) *HMOX1* were represented relative to *GAPDH*, analyzed by quantitative PCR, and expressed as fold change. (*n* = 3 independent experiments). Means followed by different letters (a, b, c) are significantly different.

To identify the impact of GMP in regulatory signals activated by cytokines, we evaluated *SOCS3* mRNA expression in activated macrophages pretreated or not with GMP (Figure 6A). The cell activation with LPS+IFN-γ increased 3.5-fold *SOCS3* expression in U937 macrophages (*p* < 0.001) in relation to control expression. Cells treated with GMP up-regulated mRNA expression of *SOCS3* 2.5-fold in comparison to activated and non-treated cells (GMP−/LPS+IFN-γ+, *p* < 0.0001) and 8.90-fold in comparison to basal expression (*p* < 0.0001).

To further explore the effect of GMP on antioxidant pathways, we evaluated *HMOX1* gene expression, which is regarded as an adaptive cellular response against inflammatory and oxidative injury [35]. As shown in Figure 6B, *HMOX1* expression did not change in the macrophage activated with LPS and IFN-γ as compared to basal expression; nevertheless, when cells were pretreated with GMP, there was a 3.5-fold increase in *HMOX1* expression. Then, antioxidant capacities of GMP might be mediated by modifying the expression of antioxidant enzymes, such as HMOX-1.

## 4. Discussion

Inflammation, oxidative stress, and dysregulated autophagy are common features in noncommunicable diseases and may be key targets for the development of novel therapeutic strategies [43]. Previous studies have shown that GMP exerts immunomodulatory, anti-oxidative, and anti-inflammatory activities in rodent models of noncommunicable diseases in which macrophage activation is related with pathophysiology [28,29,30,31]. However, the direct effect of GMP on human macrophage activation had not been studied.

The information about the effect of GMP on the macrophage activation is scarce. It has been reported that papain-hydrolyzed GMP decreases the formation of nitrites and the expression of the inflammatory genes, TNF-α, IL-1β, and iNOS, from murine macrophages RAW264.7 activated with LPS [33]. Accordingly, our results show that GMP down-regulates nitrite production and TNF-α and IL-1β secretion from U937 human macrophages stimulated with LPS and IFN-γ. Although GMP concentrations with an inhibitory effect were higher in murine than in human macrophages, the anti-inflammatory effect is equally observed. A previous study has reported that only low GMP concentrations induce U937 cell proliferation [20], which together with our results suggest that GMP exerts a modulatory activity on human macrophages at low concentrations.

GMP can be detected in blood after the intake of milk or milk-derived foods [44,45], where it may perform immunomodulatory activities. Nevertheless, ingested GMP can undergo enzymatic degradation, such as the elimination of sialic acid by the microbiota that express sialidases and use it as a carbon source [46]. Our results show that the inhibitory effect of GMP on human macrophage activation is not dependent on the content of sialic acid, suggesting that the loss of sialic acid during GMP metabolism will not affect its activity. In this sense, it is known that the Siglec-type receptors family is involved in the down-regulation of inflammatory cytokine production after sialic acid recognition [42]. Siglec-5, -7, and -9 are inhibitory receptors expressed on human macrophages under normal conditions [42,47,48]. Although their regulating role on cell activation has not been fully described, it has been reported that TNF-α production is down-regulated, while IL-10 secretion is increased in murine LPS-activated-RAW-264.7 macrophages over-expressing Siglec-9 [49]. We showed that the mRNA expression of the Siglec-7 and -9 receptors is decreased in human macrophages under the inflammatory environment induced by LPS and IFN-γ, and GMP pretreatment did not modify this condition, suggesting that neither sialic acid nor Siglec receptors are mediating the inhibitory effect of GMP on macrophage activation. Participation of sialic acid in the biological activities exerted by GMP seems to be dependent on the type of activity; while sialic acid induces protection to some viral infections [50]; a partial participation is reported on prebiotic or cell proliferation activities [20,51,52,53].

It has been widely reported that the immune response suppressor proteins, SOCS, are feedback inhibitors of the JAK-STAT pathway that can terminate both innate and adaptive responses. The SOCS3 protein is involved in the negative regulation of STAT1 and STAT3 activation, decreasing the cytokine expression [54]. We observed an increase in *SOCS3* expression in LPS and IFN-γ activated macrophages, as it has been previously reported [55]. Remarkably, human-activated macrophages pretreated with GMP showed a significantly higher up-regulation of *SOCS3* expression, which correlated with the decrease in the production of pro-inflammatory cytokines. The importance of SOCS3 in suppressing inflammatory response in macrophages has been demonstrated in mice with a conditional mutation in the *SOCS3* gene, which maintains polarization to M1 phenotype and high levels of expression of pro-inflammatory markers [55]. We theorized that *SOCS3* up-regulation induced by GMP might be involved in the down-regulating inflammatory activation of macrophages through STAT1 and STAT3 inhibition.

Studies on the antioxidant effect of GMP in hydrogen peroxide (H_2_O_2_)-activated murine macrophages RAW264.7 show that GMP induces an increase in the expression of HMOX-1 [34]. HMOX-1 is an inducible enzyme with antioxidant activities that promotes the inflammation resolution [56]. In our model of macrophage activation, LPS and IFN-γ did not increase *HMOX1* expression, but it was up-regulated when cells were GMP pretreated, which correlates with the decrease in nitrite production and pro-inflammatory cytokines. These results corroborate that GMP is an important activator of antioxidant and anti-inflammatory pathways in macrophages [33,34]. The same effect has been observed in HepG2 human liver cells after pretreated with papain-hydrolyzed GMP [57]. Moreover, up-regulation of HMOX-1 expression is also associated with beneficial effects on insulin resistance of a GMP-derived peptide in HepG2 cells [32]. Although in vivo assays are needed, based on the results of the present work, GMP might be used as a potential auxiliary therapy in the treatment of inflammation and oxidative stress-related disorders.

## 5. Conclusions

Our results demonstrate that GMP down-regulates the inflammatory environment induced by LPS and IFN-γ in human macrophages and activates anti-inflammatory and antioxidative mechanisms. Thus, by regulating macrophage activation, GMP has the potential to help in the management of metabolic alterations where cellular environments with high inflammation and oxidative stress are generated, such as noncommunicable diseases.

## Figures and Tables

**Table 1:** Primers used in this study.

Target	Oligonucleotide	Accession Number (NCBI)
*SIGLEC5*	Fw: TGTGCCCTGCTCCTTCTCTTA	NM_003830.3
	Rv: TTCCCGTGTCCTCCATTCTG	
*SIGLEC7*	Fw: TGACCACGAACAGGACCATC	NM_014385.3
	Rv: TCAACAGCACAGACCAAGCG	
*SIGLEC9*	Fw: GGCGGAAGGACAGACAAGTA	NM_001198558.1
	Rv: GCATCCTGGTCTGTATTGGC	
*SOCS3*	Fw: CACAAGAAGCCAACCAGGAG	NM_003955.4
	Rv: CTTGTGGTTGCTATCGTCCC	
*HMOX1*	Fw: AAGACTGCGTTCCTGCTCAAC	NM_002133.3
	Rv: AAAGCCCTACAGCAACTGTCG	
*GAPDH*	Fw: ATCCCATCACCATCTTCCAG	NM_002046.6
	Rv: GGCAGAGATGATGACCCTTT	

Fw, forward; Rv, reverse.

**Table 2:** Percentage of cell viability.

LPS/IFN-γ	GMP	aGMP
100 ± 0.03%	88.03 ± 5.26%	94.13 ± 3.65%

## Data Availability

All related data are presented in this paper and are available upon request to the corresponding authors.
